# Addition of Olive Leaf Extract (OLE) for Producing Fortified Fresh Pasteurized Milk with an Extended Shelf Life

**DOI:** 10.3390/antiox8080255

**Published:** 2019-07-30

**Authors:** Rosa Palmeri, Lucia Parafati, Daniela Trippa, Laura Siracusa, Elena Arena, Cristina Restuccia, Biagio Fallico

**Affiliations:** 1 Di3A, Dipartimento di Agricoltura, Alimentazione e Ambiente, University of Catania, via S. Sofia 100, 95123 Catania, Italy; 2 CNR-ICB, Consiglio Nazionale delle Ricerche-Istituto di Chimica Biomolecolare, via Paolo Gaifami 18, 95126 Catania, Italy

**Keywords:** α-glucosidase inhibition, *Bacillus cereus*, mesophilic bacteria, nutrient losses

## Abstract

An olive leaf extract (OLE) has been tested in vitro for its antibacterial activity and ability to inhibit α-glucosidase enzyme. OLE was also evaluated for its potential, when added to pasteurized milk, to preserve nutritional parameters and to limit microbial growth, thus prolonging shelf life. In vitro assays demonstrated a strong antibacterial efficacy of OLE mainly against *Bacillus cereus* and the capacity to inhibit α-glucosidase enzyme (IC_50_) when used at 0.2 mg oleuropein/mL. The milk fortification with OLE at 3.6 mg of oleuropein/mL of milk reduced total mesophilic bacteria at undetectable level after 6 d (expiration date) and by 1 log CFU/mL after 10 d. Moreover, OLE addition at 1.44 and 3.6 mg of oleuropein/mL of milk significantly reduced fat and lactose losses up to 10 d. The results motivate the use of the OLE to make a new functional milk with an extended shelf life.

## 1. Introduction

Milk is an important source of nutrients and energy, containing valuable macro and micronutrients as carbohydrates, proteins, fat, minerals, and vitamins.

Treatments that use high temperatures, such as ultra-high temperature (UHT), allow to extend milk shelf life up to 6–12 months when stored at ambient temperatures [1,2,3] but can damage the biological properties of its components. On the contrary, pasteurized milk, subjected to a thermal treatment with high temperature for short time (HTST), is a product of excellent nutritional quality but with a shelf life of only 6 days at cold storage and relatively expensive if compared to the UHT milk.

Quality of pasteurized milk during storage can be affected by the presence of spoilage bacteria, mainly spore-forming species, such as *Bacillus cereus* [4], which is able to produce proteinase, lipase, and phospholipase enzymes thus originating off-flavors (“sweet curdling” and “bitty cream” defects).

Previous studies investigated the in vitro antimicrobial activity of the olive leaf extract (OLE) or its phenolic compounds against a wide range of bacteria, including *B. cereus* [5,6], and its efficacy in extending the shelf life of food [7,8,9].

In addition, the phenolic substances present in olive leaves, mainly oleuropein [10], possess many human health benefits [11,12,13], including the capacity to prevent the fast breakdown of sugar and to control blood sugar level, therefore representing a tool for management of postprandial hyperglycemia, in particular by the inhibition of α-glucosidase activity [14].

To the best of our knowledge, no investigations have been carried out on the antimicrobial activity of OLE in pasteurized milk and on its inhibitory effect on α-glucosidase activity.

Therefore, the purpose of the study was (i) to screen in vitro the antibacterial activity of OLE against one of the major spoilage organisms of processed milk as *B*. *cereus*, and against the Gram negative *Salmonella enterica* and *Escherichia coli*; (ii) to assess the ability of OLE to inhibit in vitro α-glucosidase enzyme, so acting as a natural antidiabetic remedy; (iii) to evaluate the effectiveness of OLE as functional ingredient both on microbiological and chemical quality parameters of pasteurized milk during its shelf life.

## 2. Materials and Methods

### 2.1. Olive Leaf Extract Preparation

Organic olive leaves cultivar Nocellara were collected during the pruning period (February–March). Leaves were dehydrated at 40 °C until constant weight and subsequently used to prepare an aqueous extract (5 g of dry leaves homogenized in 100 mL of distilled water at 80 °C), as reported by Palmeri et al. [11]. The OLE obtained was filter-sterilized using a 0.20 µm pore-size membrane filter and stored in the dark at −20 °C until use.

### 2.2. Chemicals and Reagents

All solvents and reagents used in this study, Folin–Ciocalteu’s phenol reagent, sodium carbonate (Na_2_CO_3_), 2,2-Diphenyl-1-picrylhydrazyl (DPPH), ethanol (≥96%), sodium hydroxide (NaOH), sulfuric acid (H_2_SO_4_), amyl alcohol, Fehling′s A solution (CuSO_4_), and Fehling′s B solution (KOH and Na-K tartrate), were of analytical grade and purchased from Sigma–Aldrich (Milan, Italy). HPLC grade water, acetonitrile, and methanol were obtained from VWR (Milan, Italy). Pure luteolin, luteolin 7-*O*-glucoside, and apigenin-7-*O*-glucoside were provided by Extrasynthese (Lyon, France). Rutin (quercetin 3-*O*-rutinoside), apigenin, caffeic acid, chlorogenic acid, *p*-coumaric acid, ferulic acid, hydroxytyrosol, oleuropein, and 3,4-dihydroxyphenilacetic acid (DOPAC) were obtained from Fluka (Sigma-Aldrich, Milan, Italy).

### 2.3. Qualitative and Quantitative Determination of OLE Polyphenols by HPLC/DAD and HPLC/ESI-MS

High performance liquid chromatographic analyses were carried out on an Ultimate3000 instrument equipped with a binary high-pressure pump, a Photodiode Array detector, a Thermostated Column Compartment, and an Automated Sample Injector (Thermo Scientific, Milan, Italy). Collected data were processed through a Chromeleon Chromatography Information Management System v. 6.80. Chromatographic runs were performed using a reverse-phase column (Gemini C_18_, 250 × 4.6 mm, 5 μm particle size; Phenomenex, Torrance, CA, USA) equipped with a guard column (Gemini C_18_ 4 × 3.0 mm, 5 μm particle size; Phenomenex). OLE polyphenols were eluted according to Gambacorta et al. [15]. The diode array detector (DAD) was set in the range between 190 and 600 nm, recording the chromatograms at 280, 330, and 350 nm. HPLC/ESI-MS analyses on OLE were performed using a Waters instrument (Waters Italia S.p.A., Milano, Italy) and the same chromatographic conditions (solvents, elution program, guard column, column, injection volume, and flow) described above; total ion current (TIC) chromatograms were acquired in negative mode, using a cone voltage of 20 V in the mass range between 100 and 1200 *m*/*z* units. The other parameters used for the acquisition of the TICs were the following: capillary voltage: 2.75 kV; source temperature: 150 °C; desolvation temperature: 280 °C; gas flow (L/h): 400 (desolvation) and 210 (cone). Collected data were processed through a MassLynx v. 4.00 software (Waters S.p.A. Milano, Italy). Quantification of hydroxytyrosol, hydroxytyrosol glucoside, ligstroside, oleuropein, and oleuropein aglycone was carried out at 280 nm using calibration curves established with oleuropein (*R*^2^ = 0.9993) and hydroxytyrosol (*R*^2^ = 0.9992), whilst DOPAC was quantified at the same wavelength using its corresponding analytical standard (*R*^2^ = 0.9997). Apigenin 7-*O*-glucoside and apigenin were quantified at 330 nm using the calibration curve established with apigenin (*R*^2^ = 0.9995); caffeic acid (*R*^2^ = 0.9998) was used to quantify caffeic acid, ferulic acid, chlorogenic acid, and verbascoside, whilst quantification of *p*-coumaric acid was done using the corresponding available standard (*p*-coumaric acid, *R*^2^ = 0.9999). Both calibration curves were built at 330 nm. Luteolin, luteolin 7-*O*-glucoside and rutin were quantified at 350 nm using calibration curves established with luteolin (*R*^2^ = 0.9999), luteolin-7-*O*-glucoside (*R*^2^ = 0.9994), and rutin (*R*^2^ = 0.9999). Analyses were always carried out in triplicate.

### 2.4. Antioxidant Capacity of OLE

Antioxidant capacity was determined using the DPPH radical scavenging activity method [16], with slight modifications. Briefly, an aliquot of 3 mL of methanol DPPH (2,2-Diphenyl-1-picrylhydrazyl) solution 100 µM was mixed with 50 µL of different concentrations of extract (1.44, 0.72, 0.288, 0.144, 0.072 mg/mL, expressed as oleuropein content) all the extracts were previously filtered as reported in Section 2.1, homogenized, and incubated in the dark for 1 h at 25 °C.

After incubation, the absorbance of each sample was spectrophotometrically measured at 515 nm using PerkinElmer lambda 25 Uv-Vis spectrometer (PerkinElmer, Milano, Italy) against a blank (methanol DPPH solution without extract sample). The radical scavenging activity (RSA) was calculated according to the following formula:

RSA %: [(Absorbance blank − Absorbance sample) / Absorbance blank] × 100

Antioxidant capacity analysis was carried out in triplicate.

EC_50_ (efficient concentration of a compound that gives half-maximal response) was also calculated on five OLE samples at 1.44, 0.72, 0.288, 0.144, and 0.072 mg oleuropein/mL.

### 2.5. In Vitro Inhibition of α-Glucosidase Activity and IC_50_ Determination

The inhibitory capacity of OLE on α-glucosidase (α-glu, EC 3.2.1.20) was assessed as described by Jabeen et al. [17] with some modifications. Briefly, 0.1 units/mL of α-glucosidase (Sigma, type III, from *Saccharomyces cerevisiae*) was dissolved in buffer A (0.1 mol/L potassium phosphate, 3.2 mmol/L MgCl_2_, pH 6.8); the substrate *p*-nitrophenyl-α-d-glucopyranoside was dissolved in buffer A at 6 mmol/L. A total of 102 μL of buffer B (0.5 mol/L potassium phosphate, 16 mmol/L MgCl_2_, pH 6.8), 120 μL of OLE at different oleuropein concentrations (0–1.44 mg oleuropein/mL), and 282 μL water were mixed and left to react at 37 °C for 10 min; then the reaction was stopped by adding 1.2 μL of 0.4 mol/L glycine buffer (0.1 M, pH 10). Enzyme activity was quantified by measuring absorbance at 400 nm. The α-glu inhibitory activity was expressed as percentage inhibition. OLE was assessed for its inhibition potential and the IC_50_ value, concentration of an inhibitor required for reducing 50% of enzyme activity, was determined. Analyses were carried out in triplicate.

### 2.6. In Vitro Evaluation of OLE Antimicrobial Activity

The antimicrobial activity of different OLE concentrations was evaluated against *B. cereus*, *Salmonella enterica*, and *Escherichia coli* (Di3A microbial collection, University of Catania), representative for milk spoilage, process hygiene indicator, and pathogenic microorganism, respectively.

Bacterial strains were cultured in Nutrient Broth (Biolife Italiana S.r.l., Milano, Italy) for 24–48 h and then inoculated to a final cell concentration of 10^6^/mL into 20 mL of melted Nutrient Agar (Biolife Italiana S.r.l.) cooled at 45 °C, gently mixed, and poured into sterile Petri plates. After solidification, wells were made on agar plates by using a sterile cork borer (5 mm diameter) and filled with 60 µL of pure (at 1.44 mg/mL oleuropein) and diluted extract (1:2 and 1:5 *v*/*v*, at 0.72 and 0.29 mg/mL oleuropein, respectively). Control plates were made filling the wells only with sterile distilled water (SDW). Plates were incubated at 30 °C (*B. cereus*) and 37 °C (*S. enterica* and *E. coli*) for 48 h. The inhibitory effect of the extract against the tested bacterial strains was assessed measuring (mm) the inhibition halo (no bacterial growth) around the well. Analyses were carried out in triplicate.

### 2.7. Preparation of OLE-Enriched Milk

Experimental milk samples were prepared by using fresh pasteurized whole milk produced by the Sicilian industry “Latte Sole s.p.a.” (Catania, Italy). Milk bottles with the same production lot and the same expiration date were bought in a local super market at the beginning of the shelf life (production date, coincident with the first day available for the consumer), transferred to the laboratories of Di3A (University of Catania) under refrigerated conditions and immediately processed. Milk was aliquoted under sterile conditions in 50 mL tubes and different volumes of OLE were added to obtain 1%, 2%, and 5% (*v*/*v*) of OLE/milk concentration, corresponding to 1.45, 2.94 and 7.57 mg of oleuropein/100 mL of milk, respectively. Tubes containing only milk were used as controls. All samples were stored under refrigerated conditions (4 ± 1 °C) until analyzed.

### 2.8. Antimicrobial Activity of OLE on Whole Pasteurized Milk

Antimicrobial properties of OLE added to milk, as described before, were evaluated after 0, 6 (expiry date), 8, and 10 d of storage by estimating the total mesophilic bacteria (TMB) and the total *Enterobacteriaceae* counts. Each milk sample, added or not with OLE, was serially diluted with sterile Ringer solution (BR0052, Oxoid, Basingstoke, UK), and pour plated (1 mL) in Petri plates containing Plate Count Agar with cycloheximide 0.1% solution (to avoid fungal growth) and Violet Red Bile Glucose Agar (Oxoid, Basingstoke, UK), respectively for the two microbial groups. The plates were incubated at 32 °C for 24–48 h. Bacterial colonies were counted, and the mean was expressed as log10 CFU (colony forming unit)/mL of milk ± the standard error. Analyses were carried out in triplicate.

### 2.9. Compositional and Color Analysis of Milk Samples Enriched with OLE

The influence of OLE on milk quality was evaluated by analyzing the main quality parameters during refrigerated storage, such as total protein, lactose, and fat contents.

Protein (expressed as total nitrogen content) and fat were determined using standard AOAC methods [18]. The repeatability of the methods were within the acceptable values [18]. The content of lactose was analyzed by using the Fehling’s solution method [19]. Analyses were carried out on samples containing 0%, 1%, 2%, and 5% of OLE in milk after 0, 6, and 10 days of refrigerated storage (4 ± 1 °C). Within the same sample treatment, the losses of protein, lactose, or fat content were expressed as the difference between the values recorded at time 0 and those after 6 or 10 days of storage. Analyses were carried out in triplicate.

The color of milk samples, both enriched (1%, 2%, and 5% OLE *v*/*v*) and not enriched, was measured using a portable colorimeter Konica Minolta CM-2500d (Bremen, Germany) equipped with a Light Protection Tube with plate 40 mm (CR-A33b) and a sample cell made of optical glass (Glass Cell CR-A504), using as illuminant D65.

CIELAB space parameter, as Lightness (L *), redness (a *), and yellowness (b *) and psychometric correlates of chroma and hue angle, were determined at time 0 and after 6 and 10 d at 4 ± 1 °C on milk previously mixed before each determination.

The chroma (*C*) and the hue angle (h) were calculated using following equations:

(1) $$C=\sqrt{\left(a{*}^{2}+b{*}^{2}\right)}$$

(2) $$h=tan^{-1}\frac{b_{*}}{a_{*}}$$

The hue angle was expressed as Δh variation within each sample at time 0 and after 6 days of refrigerated storage.

Moreover, the color differences (Δ*E*) among milk with different amount of OLE (1%, 2%, and 5%) were calculated after 0 and 6 days of refrigerated storage using the following equation:

(3) $$\Delta E=\sqrt{{\left(L_{x}-L_{0}\right)}^{2}+{\left(a_{x}-a_{0}\right)}^{2}+{\left(b_{x}-b_{0}\right)}^{2}}$$

where subscript “x” indicates the color of milk with 1%, 2%, or 5% OLE and “0” indicates the color of milk without OLE.

Analyses were carried out in triplicate.

### 2.10. Sensory Analysis

A total of 100 subjects, 60 females and 40 males between 22 and 60 years of age, were recruited among students and staff of the University of Catania. Subjects were selected on the basis of their frequency of milk consumption (at least once a week) and the absence of food allergy or intolerance related to milk consumption. Participants were told that the aim of the study was to evaluate the hedonic liking of milk samples and, before the beginning of the tasting session, they signed the informed consent according to the principles of Declaration of Helsinki.

The tasting session was conducted in an equipped laboratory [20] in individual booths illuminated with white light. Milk samples (0%, 1%, 2%, and 5% of OLE content) at time 0, were served in disposable plastic cups in randomized order and after tasting consumers rated their liking degree on a 9-point hedonic scale, which ranged from 1 ‘‘dislike extremely” (left end) to 9 “like extremely”(right end) and at the central ‘‘neither pleasant nor unpleasant” (5 points).

### 2.11. Statistical Analysis

Data collected were analyzed using the Statistical package software Minitab^TM^ version 16.0. Differences between experimental groups were determined, on mean values, with one-way analysis of variance (ANOVA) and significant (*p* < 0.05) differences (mean separation) between treatments were carried out by Fisher’s least significant difference test.

## 3. Results and Discussion

### 3.1. Identification of OLE Polyphenols

**Figure 1:**
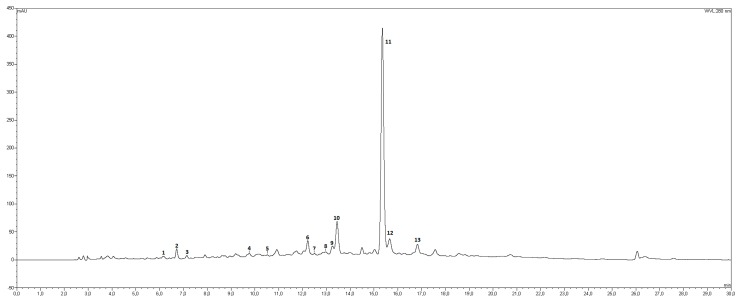
HPLC-DAD profile, visualized at 280 nm, of the olive leaf extract (OLE) object of this study. Phenolic compounds tentatively identified (see text and Table 1 for peak list and details): (1) hydroxytyrosolglucoside; (2) hydroxytyrosol; (3) DOPAC; (4) chlorogenic acid; (5) caffeic acid; (6) verbascoside; (7) p-coumaric acid; (8) ferulic acid; (9) rutin; (10) luteolin 7-*O*-glucoside; (11) oleuropein; (12) apigenin 7-*O*-glucoside; (13) ligstroside.

The HPLC/DAD chromatogram corresponding to OLE is depicted in Figure 1 (λ = 280 nm). Numerous papers present in the literature report the detailed composition of olive leaves extracts from various cultivars/regions/countries ([21] and the references therein); the phenol hydroxytyrosol and its derivatives, including oleuropein, are considered a sort of “trademark” of this matrix as well of that of the main *Olea europaea* product, olive oil. As part of the studies carried out on the still unexploited potential of olive leaves, the phenolic profile of an aqueous extract obtained from the leaves of the Sicilian cultivar Biancolilla was recently studied for biotechnological purposes [22], thus finding oleuropein and hydroxytyrosol glucoside as the main compounds.

In the present work, OLE from the Sicilian cultivar Nocellara was analyzed over the about 30 peaks appearing in the chromatogram, 13 signals were tentatively identified by comparing their relative retention times and spectral data (Uv-vis and MS) with those of corresponding analytical standards when available; assignments were further corroborated by literature data (Table 1). All compounds identified in OLE belong to the class of polyphenols, as broadly reported in literature; oleuropein (peak 11, Figure 1) clearly dominates the chromatographic profile as well as the total polyphenols content with 28.86 mg/g dry vegetable material, corresponding to 89.60%, nearly 90% of total phenolic amount (Table 1). Ligstroside (peak 13, 1.12 mg/g dry veg. mat.) and luteolin 7-*O*- glucoside (peak 10, 0.81 mg/g dry veg. mat.) were the second and third most abundant compounds, respectively; flavonoids rutin and apigenin 7-*O*- glucoside (peak 9, 0.31 mg/g and peak 12, 0.32 mg/g dry veg. mat.) were also detected and identified in OLE, together with hydroxytyrosol (peak 2, 0.20 mg/g dry veg. mat.) and its glucoside (peak 1, 0.16 mg/g dry veg. mat.). A series of hydroxycinnamic acids (peaks 4, 5, 7, 8 and their close derivative verbascoside, peak 6) were also found to belong to the secondary metabolic pool of OLE, even in small amounts Olive leaves composition may vary depending on environmental and genetic factors such as cultivar [23,24]; extraction solvent also play a pivotal role, together with extraction time and temperature [25]. Nevertheless, oleuropein as most abundant compound seems to be a recurrent data in literature, as reported by Edziri and co-workers for Tunisian *O. europaea* varieties [26], by Olmo-Garcia et al. for olive leaves collected in Morocco [27] and by Romani and others for olive leaves obtained in Italy (Tuscany, Latium, Apulia) [28]. The findings here reported, which are rather different from those of the cultivar “Biancolilla” previously studied in our laboratories, further support literature data.

### 3.2. Antioxidant Activity of Olive Leaves Extract

The radical scavenging activity (RSA%) of Nocellara aqueous OLE showed a high value 90.02 ± 0.19, comparable with that reported by Palmeri et al. [11] of 90.31 ± 0.08, for the same cultivar despite the different oleuropein content of 46.25 mg/g of dried leaves.

The extract showed an EC50 value of 0.2 μg/mL, with a regression coefficient of 0.9488.

### 3.3. Inhibition of α-Glucosidase

**Figure 2:**
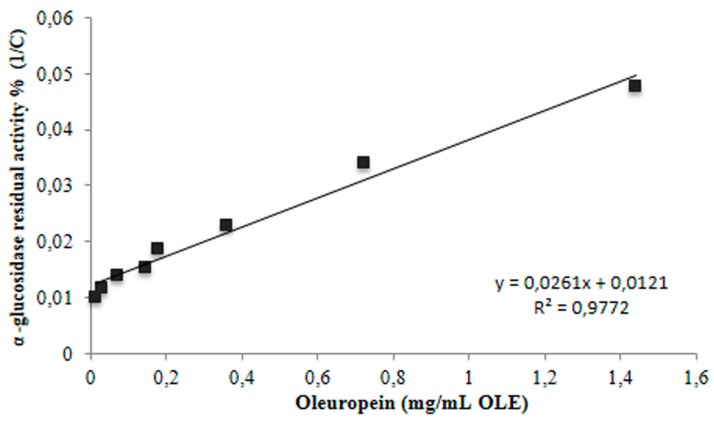
Inhibition of α-glucosidase (IC_50_) at different OLE concentrations.

The α-glu inhibitory activity was measured in the range between 0 and 2 mg polyphenols/mL OLE. As shown in Figure 2, the extract displayed an inhibitory effect with IC_50_ value at 0.2 mg oleuropein/mL of leaf extract (corresponding to a second kinetic order, R^2^: 0.977), comparable to that reported by Dekdouk et al. [29] for the standard oleuropein (IC_50_ 0.177 mg/mL) and lower than that detected for the commercially available inhibitor acarbose (IC_50_ 0.34 mg/mL). The inhibitory effectiveness of OLE on α-glu is probably related to the high content of polyphenols, oleuropein representing the most abundant compound (89.60% of total polyphenols, Table 1). The observed results are in accordance with previous studies investigating the role of phenolic compounds from different natural sources, including olive leaves, on α-glu inhibition [14,17,30]. Jabeen et al. [17] reported a large range of IC50 values for α-glu inhibition (from 11.9 to 6756.7 μM) from heterocyclic compounds. While Hadrich et al. [14] compared the α-glu inhibition from hydroxytyrosol and oleuropein respect to acarbose; in their study the IC50 of pure oleuropein was higher than acarbose, 400 and 200 μM, respectively. Our results showed a lower IC50 of OLE respect to acarbose, probably due to the synergic effect of different compounds present in the extract. The potential of phenolic compounds to affect postprandial glycemic responses by reducing glucose absorption, and thus decreasing the glycemic response of foods, when consumed together, has also been demonstrated [31,32].

### 3.4. In Vitro Antibacterial Activity of OLE

**Figure 3:**
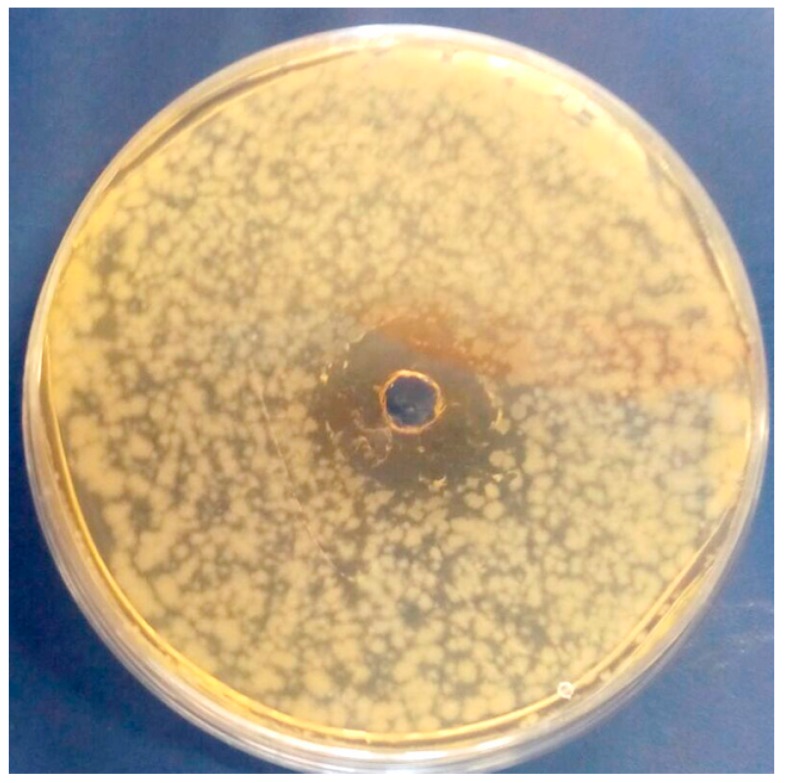
Antibacterial effect of olive leaf extract (OLE) in Petri plates inoculated with *Bacillus cereus*.

Data from the agar well diffusion assay revealed evident inhibition halos in plates inoculated with *B. cereus* (Figure 3). A weak growth inhibition effect (<2 mm) was recorded against both *S. enterica* and *E. coli*, in accordance with previous studies by Albertos et al. [8], only when undiluted OLE, at 1.44 mg/mL oleuropein, was tested; no antibacterial effect toward such Gram negative species was detected with OLEs at 0.72 and 0.29 mg/mL oleuropein (1:2 and 1:5 *v*/*v*).

In particular, *B. cereus* revealed a lack of growth both with undiluted (at 1.44 mg/mL oleuropein) and diluted (1:2 *v*/*v*, at 0.72 mg/mL oleuropein) extracts, showing inhibition halos width of 6.75 ± 0.31 and 5.33 ± 0.17 mm, respectively. *B. cereus* sensitivity towards aqueous OLE extract has been already evaluated by Pereira et al. [33]. Antibacterial activity of OLE against *B. cereus* is partially due to the presence of phenolic compounds as oleuropein, hydroxytyrosol, tyrosol, etc. [5,6,34], although the synergic effect of OLE compounds on antimicrobial activity is still not well known.

### 3.5. Antimicrobial Effect of OLE on Milk Samples

**Figure 4:**
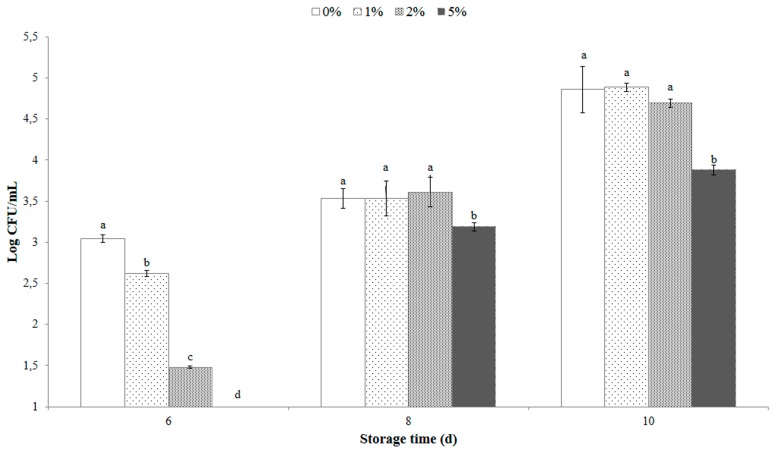
Total mesophilic bacterial (TMB) counts of milk samples added with different amounts of olive leaf extract (OLE), over 10 d of storage at refrigerated condition. Columns at the same storage time (6, 8, and 10 d) marked by different letters are significantly different according to Fisher’s least significant difference test (*p* < 0.05). Vertical bars indicate the standard deviation of the mean.

Total *Enterobacteriaceae* count in pasteurized milk added or not with OLE was below the detection limit of the plate count method throughout the considered storage period (10 d). Figure 4 illustrates the TMB counts of milk samples treated with different amounts of extract, over 10 d of storage at refrigerated condition. An antimicrobial effect of OLE was observed, more effective with increasing concentrations. In particular, the starting TMB count (time zero), was observed to be below the detection limit of the plate count method for all samples. After 6 days of storage (expiration date of milk) total count was 3.04 ± 0.02, 2.62 ± 0.04, 1.48 ± 0.01, and 0.00 ± 0.00 log CFU/mL for the samples containing OLE at 0%, 1%, 2%, and 5%, respectively. These values increased steadily during storage, reaching 3.53 ± 0.12, 3.53 ± 0.21, 3.61 ± 0.18, and 3.19 ± 0.05 log CFU/mL after 8 days of storage in samples containing 0%, 1%, 2%, and 5% of OLE, respectively. The highest extract concentration (5%) showed a statistically significant (*p* < 0.05) antimicrobial effect also after 10 d of storage (3.88 ± 0.06 log CFU/mL) in comparison to the other treatments (4.86 ± 0.28, 4.88 ± 0.05, and 4.69 ± 0.05 log CFU/mL, respectively for 0%, 1%, and 2% of OLE) (Figure 4). Furthermore, considering European Council Directive 92/46/EEC [35] that fixes a 5 × 10^4^ (4.7 log) limit for plate count at 21 °C (per mL of pasteurized milk), after 10 d storage at 4 °C, the only milk sample that fully complies with the mentioned limit is the milk sample added with 5% OLE. The addition of OLE at 2%, however, maintained the mesophilic bacterial count of milk slightly below the European limit (4.69 ± 0.05 log CFU/mL).

To the best of our knowledge no studies investigated the antibacterial effect of OLE on milk matrix, although selected bioactive compounds from *O. europea* have been proven to reduce artificially inoculated *S. enterica* on cut green leaf products [36] and to prolong shelf life of meat products [7]. In addition, active packaging with antioxidant properties have been recently developed with the inclusion of olive leaf extract [37].

### 3.6. Determination of Principal Compositional Parameters of OLE-Enriched Milk

**Figure 5:**
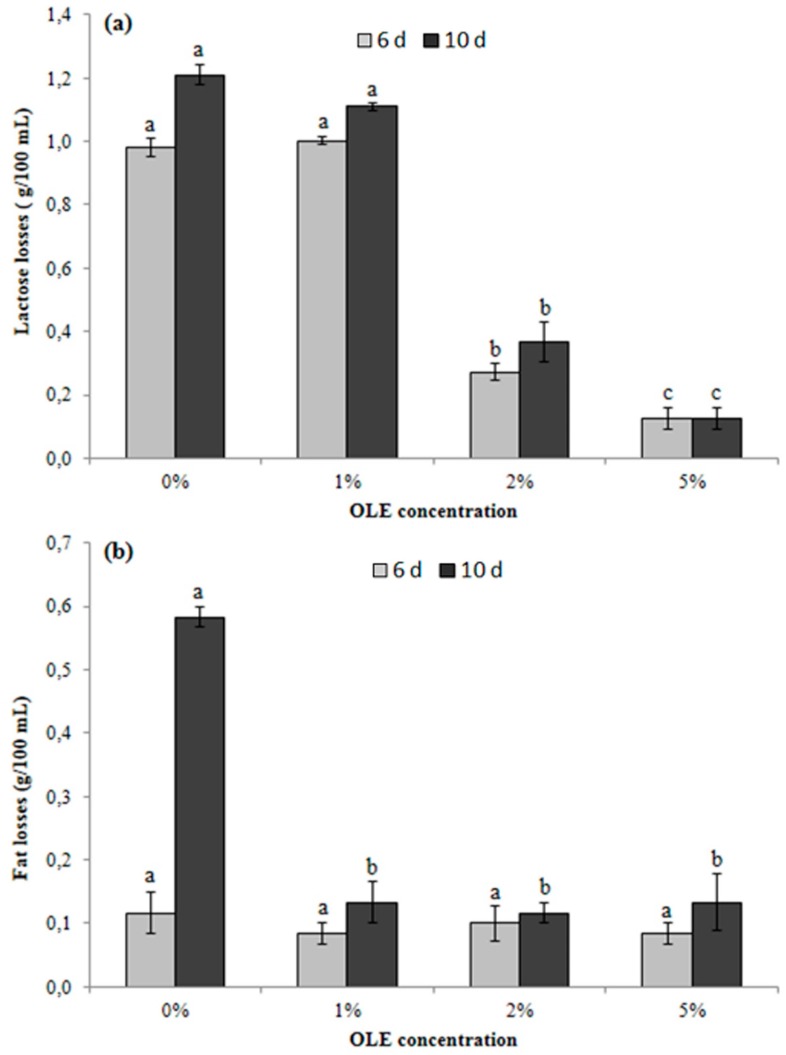
Losses of lactose (**a**) and fat (**b**) in milk samples containing 0%, 1%, 2%, and 5% of olive leaf extract (OLE) after 6 and 10 d of refrigerated storage. Bars indicate the standard error of the mean. Columns representing the same time (6 or 10 d) followed by different letters are significantly different according to Fisher’s least significant difference test (*p* < 0.05).

Regarding the protein content, no significant (*p* < 0.05) differences were recorded over the storage period (10 d; data not shown). However, since the Kjeldhal method allows to calculate the total protein content through the determined total nitrogen content, independently from protein (casein) degradation, the total N levels remain unchanged. Figure 5a,b displays the losses of lactose and fat (g/100 mL of milk) within the same sample (0%, 1%, 2%, and 5% of OLE), after 6 and 10 d of storage.

In detail, lactose losses (Figure 5a) were significantly lower (*p* < 0.05) in milk sample containing 2% and 5% of OLE than in unfortified milk (0% OLE), both after 6 and 10 d of storage. In fact, OLE addition at 2% reduced lactose losses by 70% and 67%, respectively after 6 and 10 d, and OLE addition at 5% reduced lactose losses by 90% and 92%, respectively after 6 and 10 d. The lowest lactose losses in milk containing OLE at 5% are probably connected to the extract antibacterial effect; it is well known, in fact, that most Gram negative psychrotrophic bacteria and *Bacillus* spp. have the ability to hydrolyze this sugar [38].

Different amounts of extract added to milk did not have a notable effect on fat content over 6 d of storage. Only at the end of storage period (10 d), fat losses (Figure 5b) were significantly lower (*p* < 0.05) in samples added with 2% and 5% OLE than in unfortified milk (0% OLE), with reductions of 83% and 82%, respectively. Such observation suggests a beneficial effect on lipid oxidation, as already observed in other food products [39,40,41], as well as an antibacterial effect, mainly toward *Pseudomonas* spp., which could exhibit the highest lipolytic activity in refrigerated milk (4–7 °C) [42].

### 3.7. Color

The space values L *, a *, and b *, determined both on milk enriched or not with OLE over refrigerated storage (4 ± 1 °C), are shown in Table 2 up to 6 d since after this sampling time no appreciable differences were detected for the considered parameters.

At time 0, the L * value was significantly (*p* < 0.05) affected by the presence of different amounts of OLE, decreasing at increasing OLE concentration from 0% (control) to 5%. On the contrary, at the same sampling time, the a * and b * values increased significantly (*p* < 0.05) with the increasing of OLE concentration from 0% (control) to 5%. After 6 d of refrigerated storage (4 ± 1 °C), the L * value was not significantly (*p* < 0.05) affected by the presence of different amounts of OLE; the a * value showed an unchanged trend compared to time 0 and the b * value was higher in samples containing 5% and 2% of OLE, followed by 1% and 0% (control).

As reported by Popov-Raljić et al. [43], milk is naturally subjected to color changes during storage; in particular, L * and b * values tend to decrease in relation to storage and percentage of fat. In this study, OLE addition, at time 0, determined in the milk matrix a variation of the parameters L *, a *, and b * directly proportional to the amount of OLE added, probably due to the presence of colored substances, such as chlorophylls, in the extract. Differences among the samples containing 0%, 1%, 2%, and 5% of OLE seemed to flatten after 6 d for b * and L * values. In particular, at this time b * values were significantly (*p* < 0.05) different only in the sample containing 5% OLE, while L * values had no significant differences (*p* > 0.05) (Table 2), probably connected to the antimicrobial and antioxidant properties of the extract. Bermúdez-Aguirre et al. [44]. In fact, the milk samples reported as raw and heat treated always showed a decreasing L * value during storage, caused by physiochemical reactions and microbiological growth. Taking into consideration the ΔE at time 0, it significantly (*p* < 0.05) increased at increasing OLE concentrations (Table 2). Nevertheless, these differences were “distinct” only in the milk sample containing 5% of OLE, as reported by Francis and Clydesdale [45] who considered ΔE “very distinct” if >3, “distinct” if 1.5 < ΔE < 3 and “without perceptible differences” if ΔE < 1.5. After 6 d no significant (*p* > 0.05) differences were observed in ΔE among samples.

C values, measuring color saturation or intensity, showed significant (*p* < 0.05) differences only at time 0; after 6 d of storage, no significant differences (*p* > 0.05) were observed for all OLE-added samples, while in the control sample (milk without OLE) a decrement from 5.89 to 4.36 was observed (Table 2).

Hue angle (h) describes the relative amounts of redness and yellowness [46]; also, for Δh, no significant differences (*p* > 0.05) were observed among the control and OLE-added samples during storage (Table 2).

### 3.8. Impact of OLE Addition on Liking

The hedonic test evaluated the liking degree of the four milk products in order to highlight a sensory effect due to the addition of OLE. The results of the tasting session show that the liking score for all milk samples was similar ranging from 7.1 to 7.6 for milk and milk added with 2% of OLE, respectively. No significant differences (*p* > 0.05) in liking scores were found between milk and the other milk samples containing different levels of OLE, suggesting that the OLE addition, also at the highest level, did not modify the liking degree.

## 4. Conclusions

Olive leaf extract (OLE) effectively inhibited in vitro the growth of *B. cereus* and α-glu activity. By considering overall microbiological and nutritional results of in vivo experimental trial, the addition of 5% OLE (*v*/*v*) to whole pasteurized milk increased its shelf life by 60%, which would lead to significant benefits in terms of costs linked to transport and to product returns to the dairy industry.

Therefore, OLE may be considered a valuable potential ingredient for the creation of a fortified milk both for its preservative and functional properties.

## Figures and Tables

**Table 1:** Peak list and content of selected metabolites present in olive leaf extract (OLE). See text for details.

Peak	Compound	Rt	Content mg/mL	Content (mg/g dry veg mat)
1	Hydroxytirosol glucoside	6.16	0.008	0.16
2	Hydroxytirosol	6.74	0.010	0.20
3	Dihydroxyphenylacetic acid (DOPAC)	7.17	0.004	0.08
4	Chlorogenic acid	9.87	0.002	0.04
5	Caffeic acid	10.21	0.002	0.04
6	Verbascoside	12.33	0.013	0.26
7	p-Coumaric acid	12.43	0	tr
8	Ferulic acid	13.03	0	tr
9	Rutin	13.39	0.016	0.31
10	Luteolin 7-*O*-glucoside	13.48	0.040	0.81
11	Oleuropein	15.5	1.44	28.86
12	Apigenin 7-*O*-glucoside	15.69	0.016	0.32
13	Ligstroside	16.99	0.056	1.12

tr: traces.

**Table 2:** Color parameters of milk samples enriched with OLE during storage.

	Color Parameters	OLE Content (*v*/*v*)			
		0%	1%	2%	5%
Storage Time 0 (d)	L *	81.08 ± 0.00 ^a^	80.93 ± 0.01 ^b^	80.63 ± 0.00 ^c^	80.18 ± 0.00 ^d^
	a *	−2.54 ± 0.00 ^d^	−2.49 ± 0.01 ^c^	−2.41 ± 0.01 ^b^	−2.24 ± 0.01 ^a^
	b *	5.31 ± 0.00 ^d^	5.40 ± 0.00 ^c^	5.46 ± 0.01 ^b^	5.74 ± 0.00 ^a^
	C	5.89 ± 0.00 ^d^	5.95 ± 0.01 ^c^	5.97 ± 0.01 ^b^	6.16 ± 0.01 ^a^
			ΔE 0–1%	ΔE 0–2%	ΔE 0–5%
			0.19 ± 0.01 ^c^	0.37 ± 0.01 ^b^	1.04 ± 0.00 ^a^
Storage Time 6 (d)	L *	79.11 ± 1.39 ^a^	79.13 ± 1.53 ^a^	78.87 ± 1.55 ^a^	78.05 ± 1.59 ^a^
	a *	−2.45 ± 0.81 ^d^	−2.32 ± 0.05 ^c^	−2.21 ± 0.03 ^b^	−1.94 ± 0.03 ^a^
	b *	5.07 ± 1.10 ^b^	5.40 ± 0.13 ^b^	5.34 ± 0.14 ^ab^	5.71 ± 0.16 ^a^
	C	4.36 ± 1.29 ^a^	5.75 ± 0.14 ^a^	5.78 ± 0.14 ^a^	6.03 ± 0.16 ^a^
			ΔE 0–1%	ΔE 0–2%	ΔE 0–5%
			1.69 ± 2.28 ^a^	1.89 ± 2.11 ^a^	2.59 ± 1.69 ^a^
	Δh 0–6 ^d^	0.14 ± 0.25 ^a^	0.02 ± 0.00 ^a^	0.02 ± 0.00 ^a^	0.04 ± 0.01 ^a^

Data presented as mean ± standard error of the mean. In each row, values followed by different letter within the same parameter (L *: lightness; a *: redness; b *: yellowness, C: chroma, ΔE: color change, Δh: huge angle difference) are significantly different according to Fisher’s least significant difference test (*p* ≤ 0.05).
